# Supplementary material for: Myomerger promotes fusion pore by elastic coupling between proximal membrane leaflets and hemifusion diaphragm
Source: Nat Commun. 2021 Jan 21;12:495. doi: 10.1038/s41467-020-20804-x (PMC7820291; doi:10.1038/s41467-020-20804-x)
Supplement: Supplementary file 1 — Supplemental Materials [file 41467_2020_20804_MOESM1_ESM.pdf]

Myomerger promotes fusion pore by elastic coupling between proximal membrane leaflets and hemifusion diaphragm

Gonen Golani[1,*], Evgenia Leikina[2,*], Kamran Melikov[2], Jarred M. Whitlock[2], Dilani G Gamage[3], Gracia Luoma-Overstreet[2], Douglas P. Millay[3,4], Michael M. Kozlov[1#], Leonid V. Chernomordik[2#]

SUPPLEMENTAL MATERIALS.

Supplemental Figures 1-9

Appendices

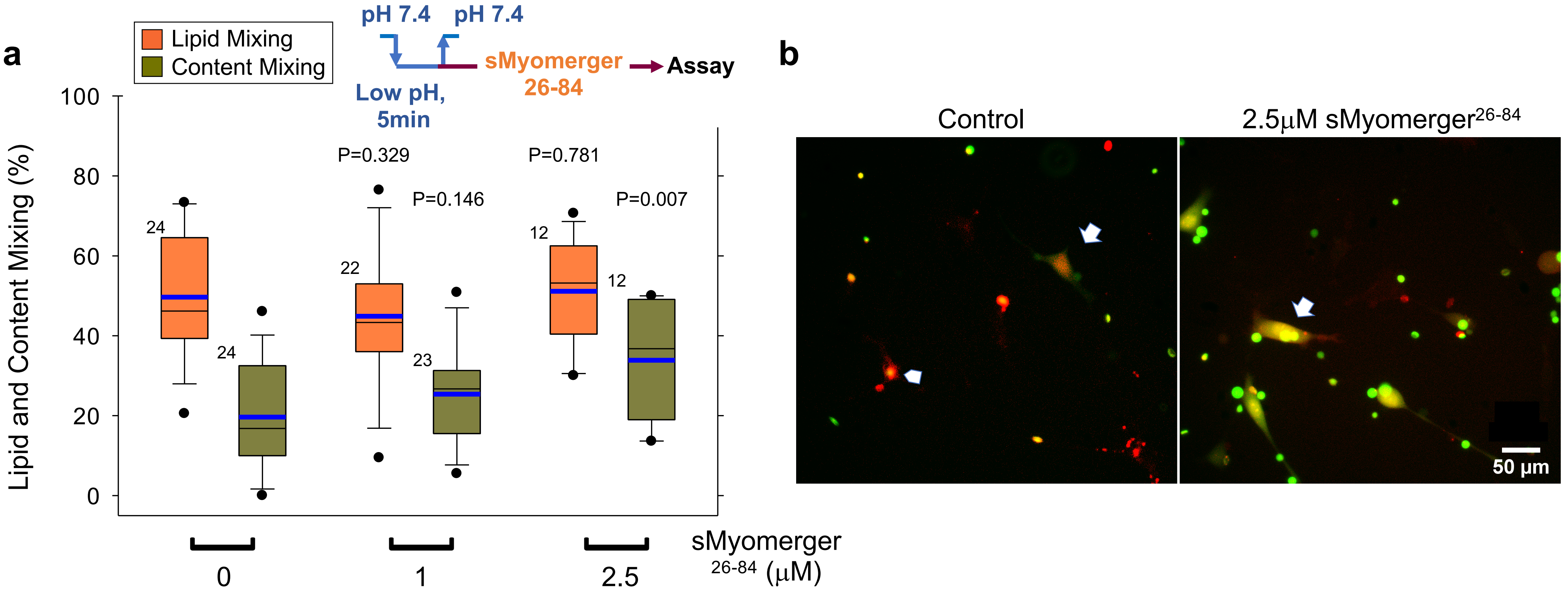

Fig. S1. **Synthetic myomerger ectodomain (sMyomerger$^{26\text{-}84}$) promotes HA-mediated fusion.** (a, b) HA-cells with pre-bound RBCs labeled with the fluorescent lipid PKH26 and loaded with carboxyfluorescein were treated with a 5-min pulse of pH 5.1 medium at 22^{0}C. Immediately after the end of the low pH pulse, we applied different concentrations of sMyomerger$^{26\text{-}84}$ for 10 min at the room temperature. (a) Fusion (redistribution of lipid and content probes from RBCs to HA-cells) was analyzed with fluorescence microscopy in ≥ 10 randomly chosen fields of view for each condition. Content mixing (fusion) and lipid mixing (fusion plus hemifusion) were quantified as the ratios of the numbers of either content probe (CF)-labeled HA-cells or lipid probe (PKH26)-labeled HA-cells, respectively, to the total number of HA-cells. Shown are representative results in one of three independent cell preparations. N, the number of randomly selected fields of view examined for each condition is indicated to the left of the box associated with that data. Box-and-whisker plots show median (center line), mean (blue line), 25th–75th percentiles (box), 10th-90th percentiles (whiskers), 5th - 95th percentiles (solid circles). P-values were calculated using a two-tailed unpaired t-test relative to the data in control (no sMyomerger$^{26\text{-}84}$). b) Representative images for the experiments in (a) taken for the control cells and for the cells treated with 2.5 µM sMyomerger$^{26\text{-}84}$. Arrows mark an example of fusion: HA-cell that acquired from fused RBC both lipid (red)- and content (green) probes.

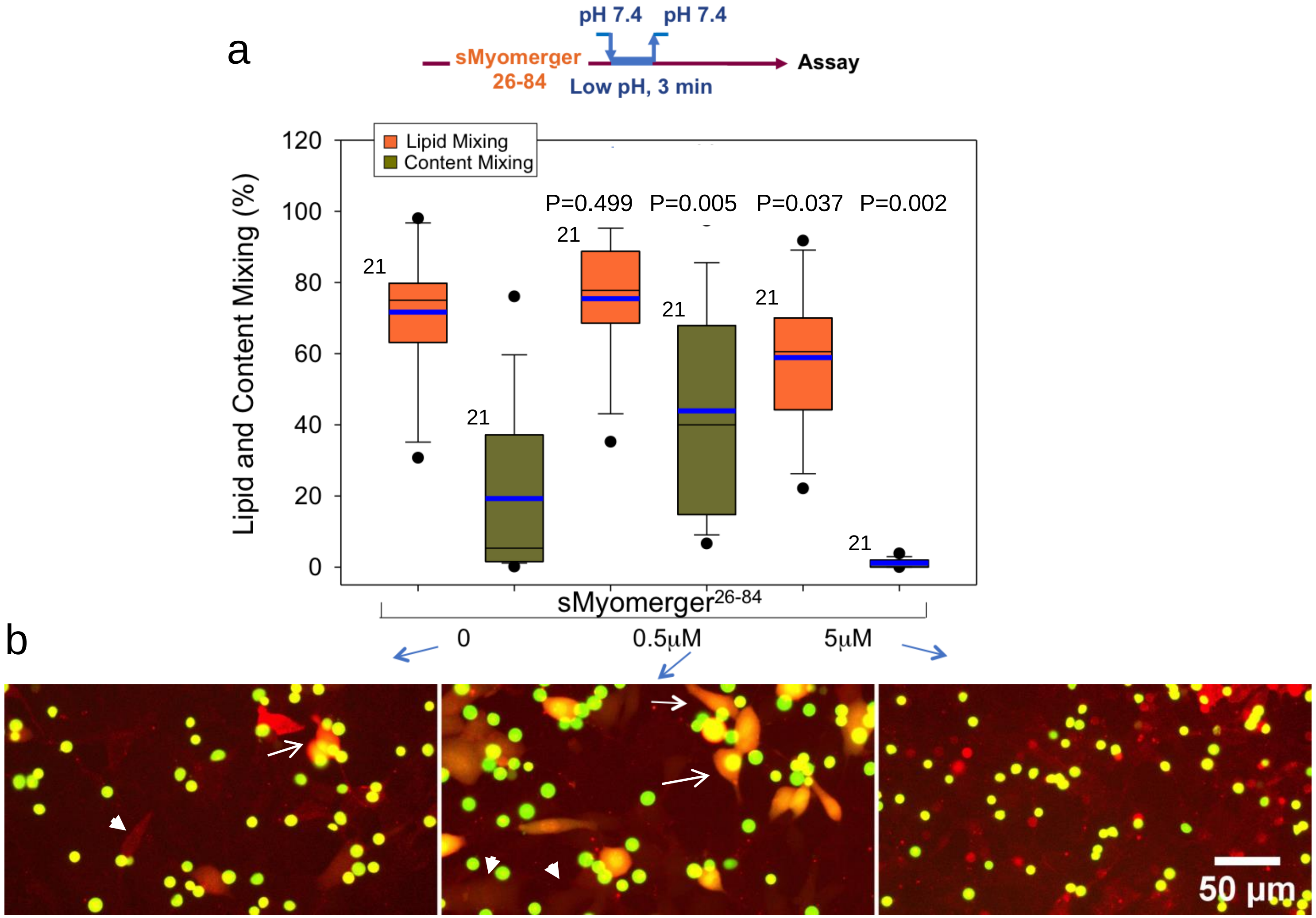

Figure S2. **Low and high concentrations of sMyomerger$^{26\text{-}84}$ applied before low pH pulse promote HA-mediated fusion and inhibit HA-mediated hemifusion and fusion, respectively.** (a) HA-cells with pre-bound RBCs were incubated for 5 min with different concentrations of sMyomerger$^{26\text{-}84}$ then treated with a 3-min pulse of pH 5.0 medium at 22^{0}C. Immediately after the end of low pH pulse, we raised the temperature to 37^{0}C. 15 min later we analyzed fusion (redistribution of lipid and content probes from RBCs to HA-cells) with fluorescence microscopy in ≥ 10 randomly chosen fields of view for each condition. Content mixing (fusion) and lipid mixing (fusion plus hemifusion) were quantified as the ratios of the numbers of either content probe (CF)-labeled HA-cells or lipid probe (PKH26)-labeled HA-cells, respectively, to the total number of HA-cells. Shown are representative results in one of three independent cell preparations. N, the number of randomly selected fields of view examined for each condition is indicated to the left of the box associated with that data. Box-and-whisker plots show median (center line), mean (blue line), 25th–75th percentiles (box), 10th-90th percentiles (whiskers), 5th - 95th percentiles (solid circles). P-values were calculated using a two-tailed unpaired t-test relative to the data in control (no sMyomerger$^{26\text{-}84}$). (b) Representative images for the experiments in (a) taken for the control cells and for the cells treated with 5 µM sMyomerger$^{26\text{-}84}$. Arrow and arrowhead mark examples of fusion and hemifusion, respectively. Scale bar 50µm.

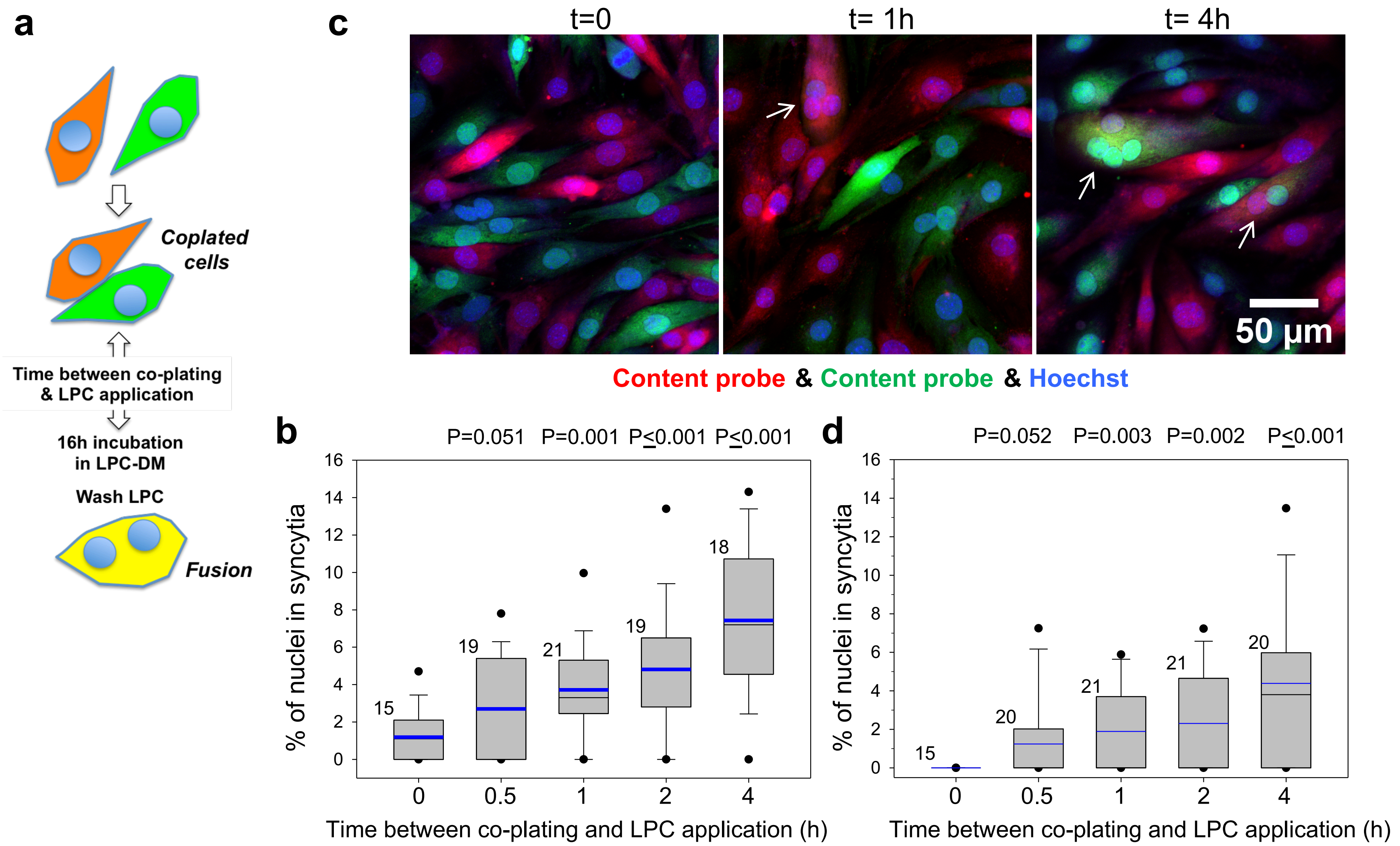

Fig. S3. **The efficiency of complete fusion between Myomerger-deficient myoblasts after LPC synchronization depends on the time between cell coplating and LPC addition.** a) Schematic showing cell fusion induced by lysophosphatidylcholine (LPC) application to co-plated differently labeled differentiating Myomerger-deficient cells. b) After 2 days in the differentiation medium (DM) we co-plated differently labeled cells and after different time intervals applied 150 μM LPC to synchronize myoblast fusion. 16 hours later LPC was washed out and 1h later we fixed the cells and scored fusion as the ratio of nuclei in the cells with ≥ 2 nuclei to the total number of nuclei. c) Representative images for the experiments in (b) taken for t=0, t=1h and 4h. Arrows mark examples of fusion. Scale bar 50mm. d) The same experiments as in (b) were analyzed counting as fused cells only double labeled cells plus cells that are not double labeled but have more than 2 nuclei. b,d) The quantifications of the fusion extents. N, the number of randomly selected fields of view examined over three independent cell preparations for each condition, is indicated to the left of the box associated with that data. Box-and-whisker plots show median (center line), mean (blue line), 25th–75th percentiles (box), 10th-90th percentiles (whiskers), 5th - 95th percentiles (solid circles). P-values were calculated using a two-tailed unpaired t-test relative to the data for t=0.

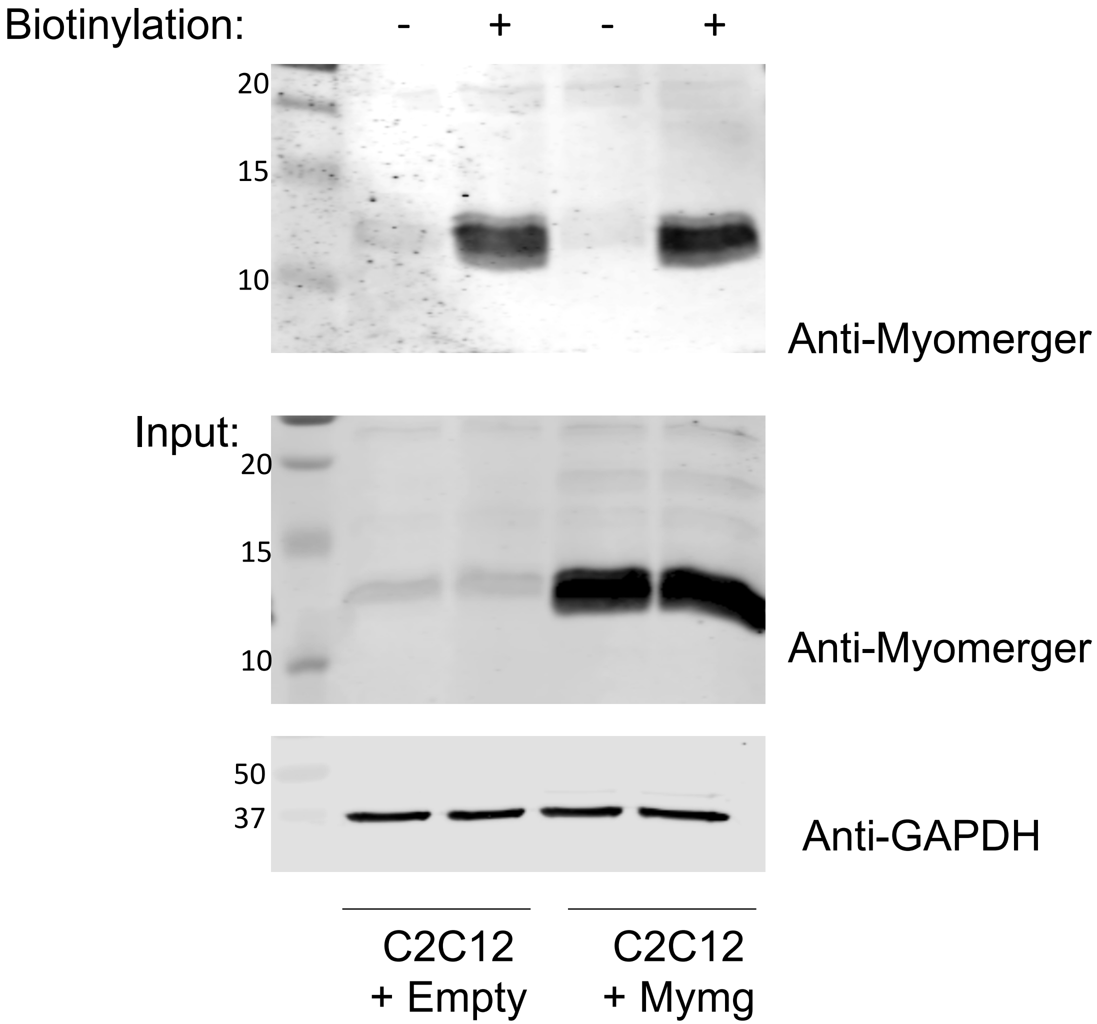

Fig. S4. **Cell surface biotinylation analysis of the cells overexpressing Myomerger.** Immunoblot for Myomerger from the surface-biotinylated fraction (IP: streptavidin) and total lysates (input) from w.t. C2C12 myoblasts transduced with empty vector or overexpressing Myomerger. Cells were differentiated for three days. Cells not treated with a cell-impermeable biotinylation reagent were used as a control. GAPDH was used as a loading control. Data shown are representative of 2 independent experiments.

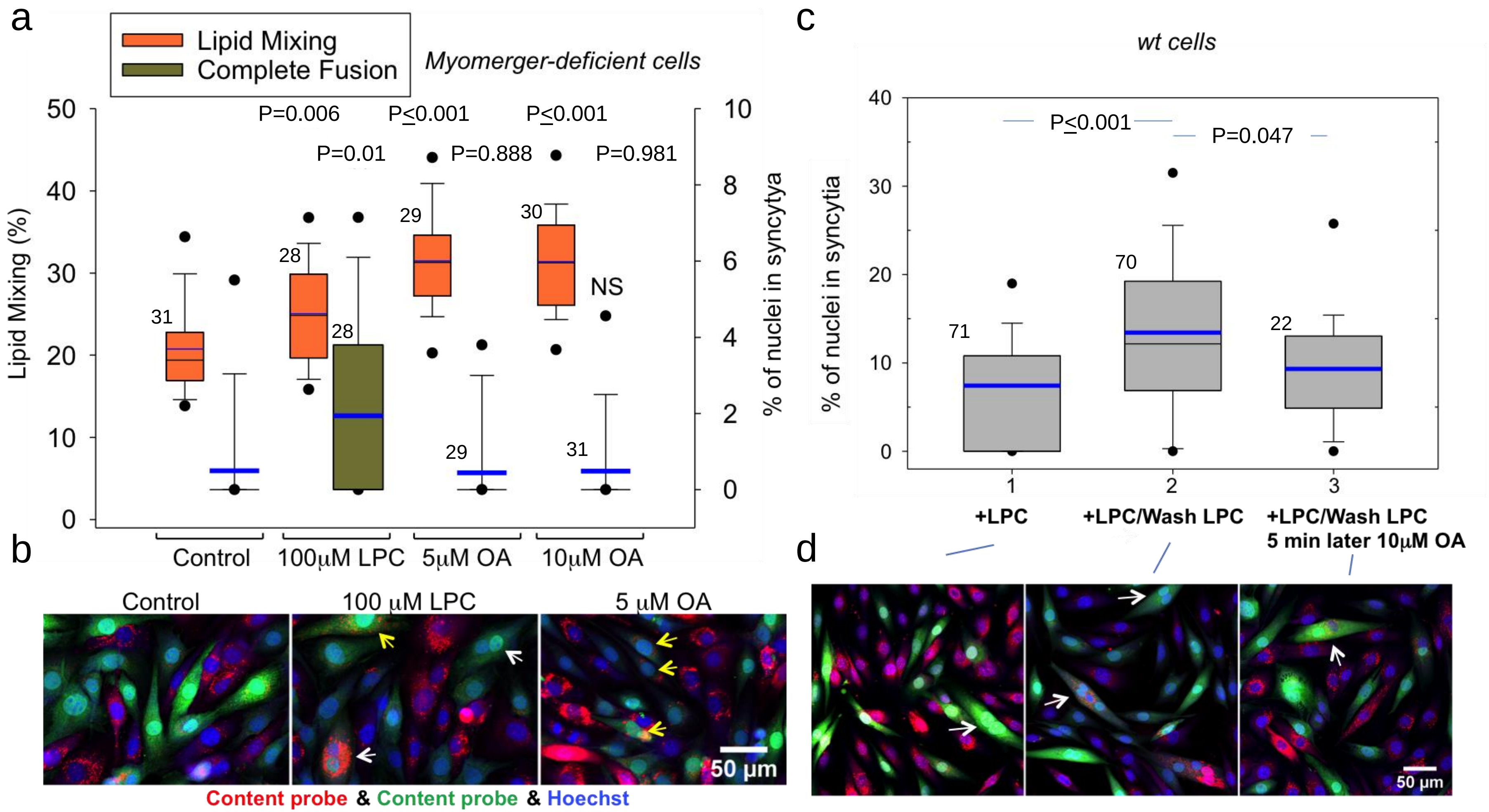

Fig. S5. **Effects of LPC and oleic acid on myoblast fusion.** (a) Myomerger-deficient cells were differentiated for 2 days, labeled either with DiI or with green cell tracker and co-plated. After 3 days of the differentiation, we applied 100 μM LPC or either 5 or 10 μM OA for 60 min. Then we fixed the cells and scored fusion as the ratio of nuclei in the cells with $\geq$ 2 nuclei to the total number of nuclei. We also scored the extents of lipid mixing by counting mononucleated cells labeled with both of our probes. (b) Representative images for the experiments in (a} taken for the control cells and for the cells treated with 100μM LPC or 5μM OA. White and yellow arrows mark examples of complete fusion and lipid mixing, respectively. Scale bar 50μm. (c) Differentiating w.t. C2C12 cells were accumulated upstream of hemifusion by the incubation in the presence of LPC. After 3 days in the DM, including last 16h in the LPC-supplemented DM, we replaced this medium with LPC-free DM (Bars 2 and 3). In Bar 3, 5 min after LPC removal we applied 10μM OA. In Bar 1 LPC was not removed. 30 min later we fixed the cells and scored fusion as the ratio of nuclei in the cells with $\geq$ 2 nuclei to the total number of nuclei. (d) Representative images for the experiments in (c). Arrows mark examples of fusion. Scale bar 50μm. (a, c) N, the number of randomly selected fields of view examined to quantify the % of nuclei in syncytia and lipid mixing extents (right and left Y-axes, respectively) in (a) and % of nuclei in syncytia in (c) over three independent cell preparations for each condition, is indicated to the left of the box associated with that data. Box-and-whisker plots show median (center line), mean (blue line), 25th–75th percentiles (box), 10th-90th percentiles (whiskers), 5th - 95th percentiles (solid circles). P-values relative to the corresponding data (lipid mixing or complete fusion) in control (a) or the data in Bar 2 (c) were calculated using a two-tailed unpaired t-test.

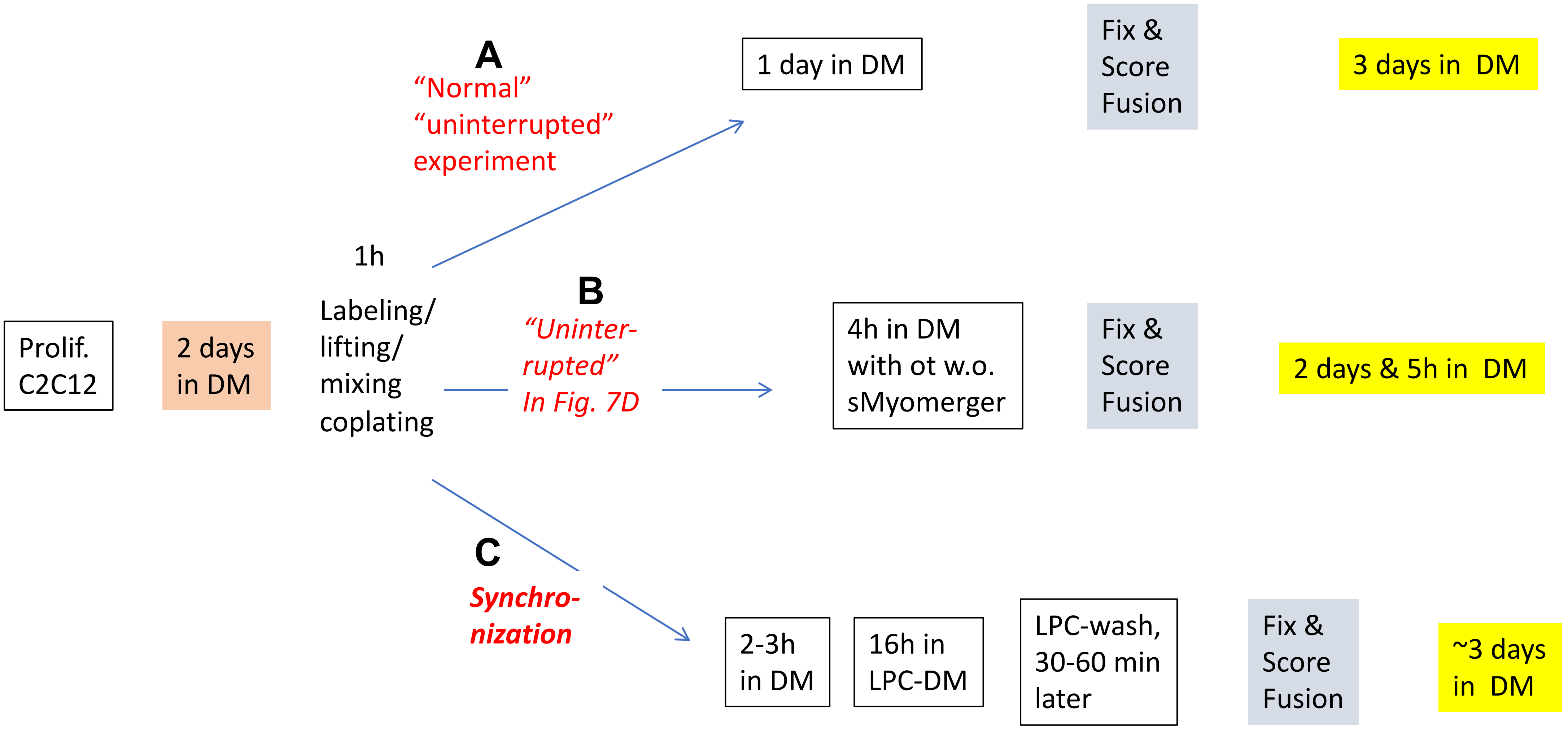

Fig. S6. Timelines of different experimental designs used in this study to prepare the cells for fusion assays (see the Methods for details).

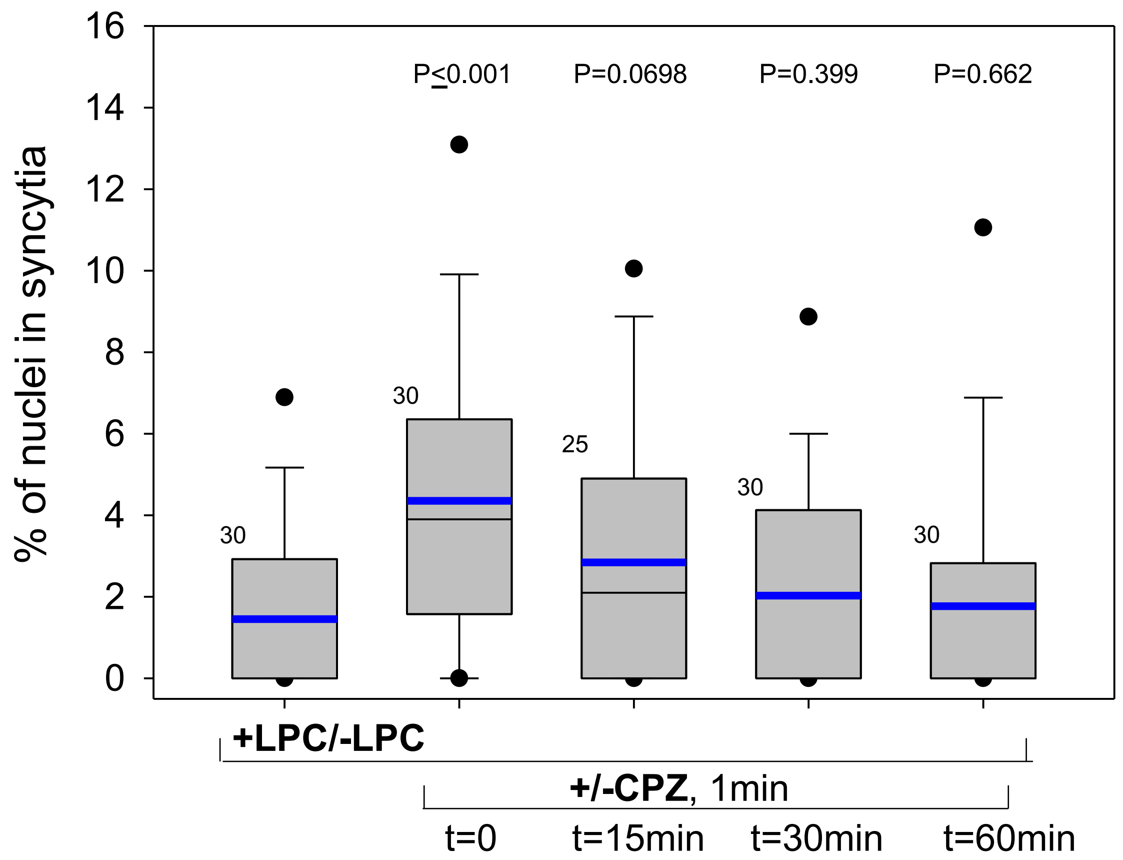

Fig. S7. **Dissociation of hemifusion connections between myoblasts assayed by counting as fused cells only double labeled cells or cells with more than 2 nuclei.** Experiments on synchronized Myomerger-deficient cells treated by chlorpromazine (CPZ) at different times after LPC removal presented in Fig. 7 were analyzed by counting as fused cells only double labeled cells and cells that are not double labeled but have more than 2 nuclei rather than all cells with ≥2 nuclei. N, the number of randomly selected fields of view examined over two independent cell preparations for each condition, is indicated to the left of the box associated with that data. Box-and-whisker plots show median (center line), mean (blue line), 25th–75th percentiles (box), 10th-90th percentiles (whiskers), 5th - 95th percentiles (solid circles). P-values were calculated using a two-tailed unpaired t-test relative to the data in +LPC/-LPC bar.

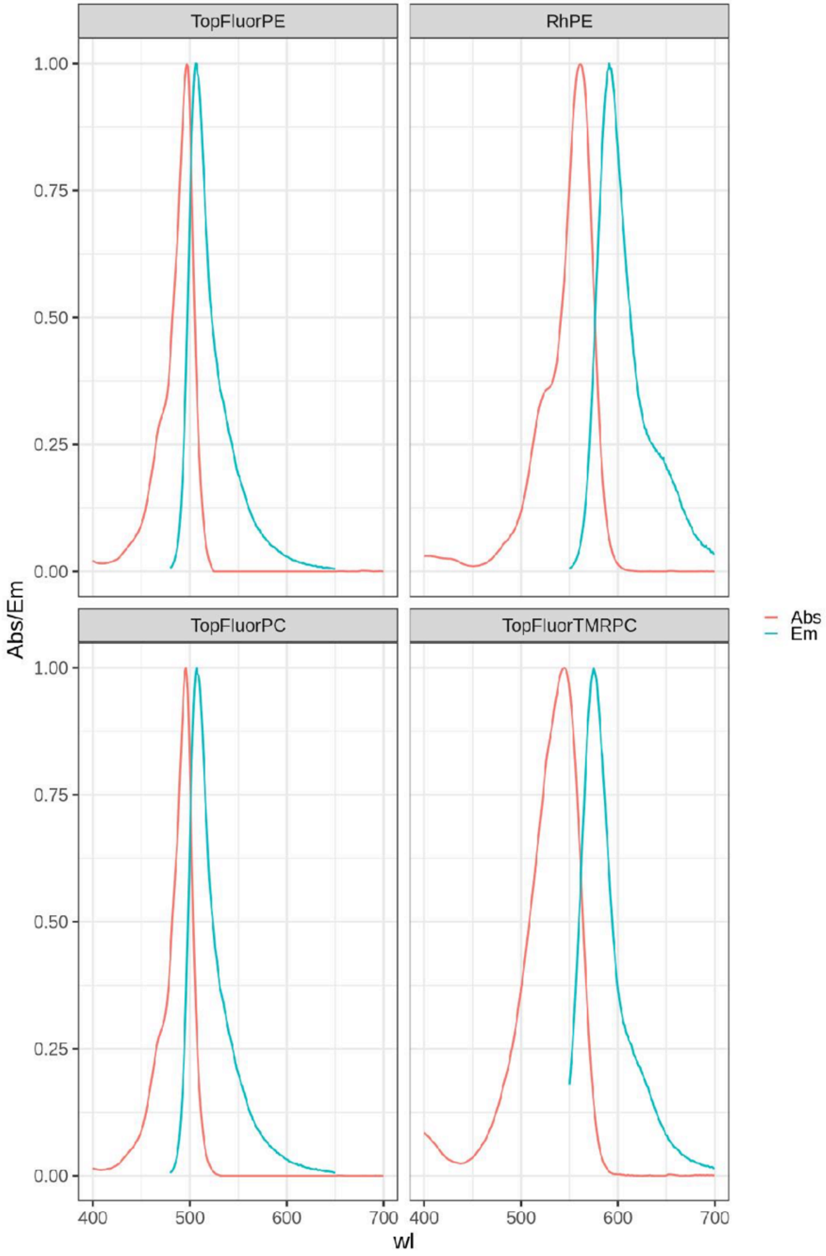

Fig. S8. **Emission and absorption spectra of individual fluorescent lipids TopFluorPE; RhPE; TopFluorPC and TopFluorTMRPC used in the FRET experiments.** Emission spectra were measured in liposomes, containing 0.5 mole percent of the corresponding fluorescent lipid, 89.5 mole percent of DOPC and 10 mole percent DOPS. Absorption spectra were measured in methanol.

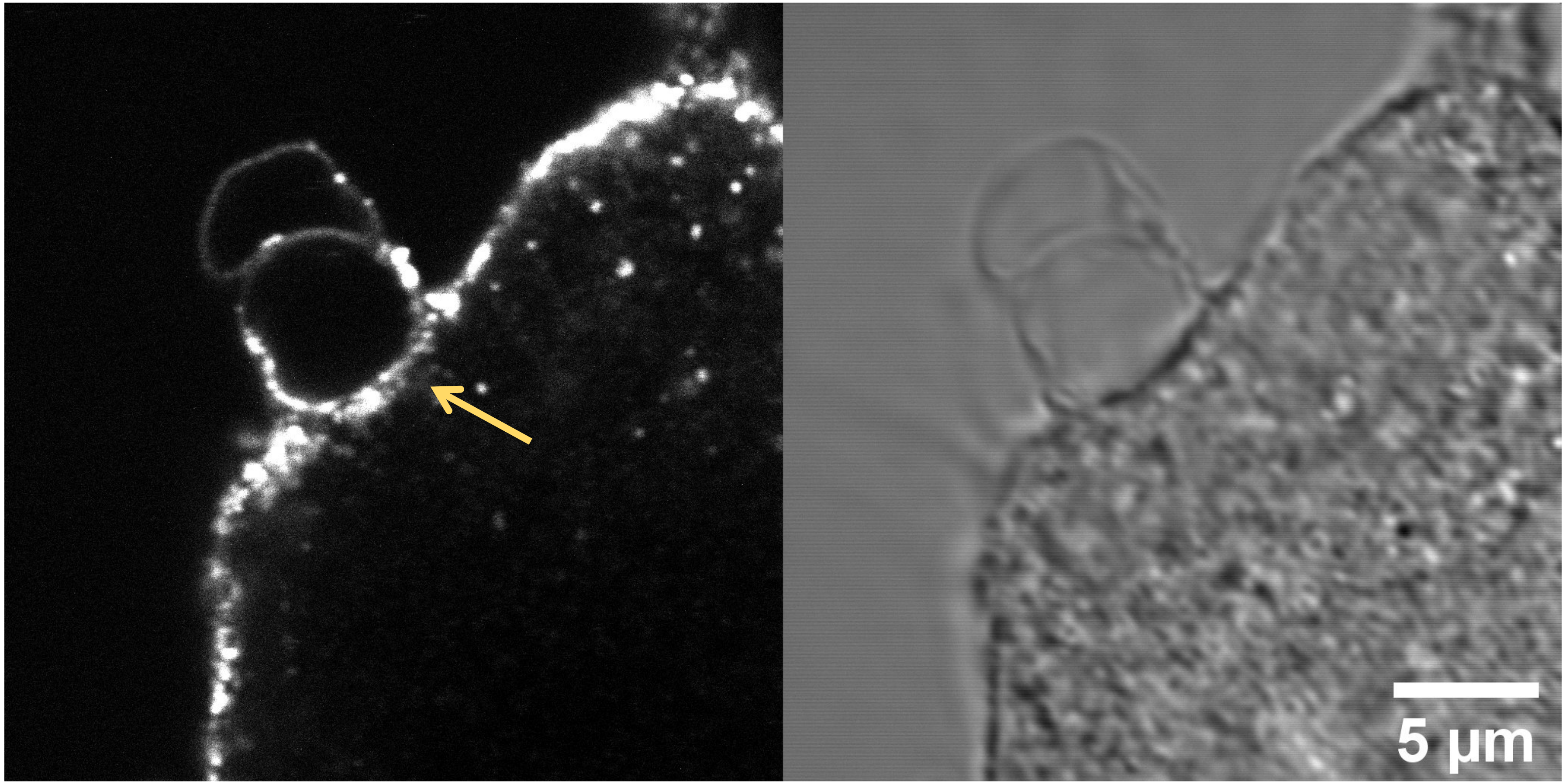

Fig. S9. **sMyomerger$^{26\text{-}84}$ readily enters the tight cell-cell contact zone.** Confocal fluorescence microscopy images (left) and phase contrast (right) of HA-cell/RBC contact (marked by arrow) taken after 5 min incubation of HA-cell/RBC complexes with TAMRA-sMyomerger$^{26\text{-}84}$ followed by washing the cells to remove unbound peptide. Shown is a representative pair of images of two independent experiments. Scale bar 5 μm.

Appendix A.

Elastic energy of the fusion site and the strategy of computations

The energy of the fusion site accounts for the elastic energy of the tilt and splay deformation of the constituent lipid monolayers, as introduced in detail in the earlier work [1-3]. In the first part of this appendix we briefly review the physical meaning of these monolayer deformations and the related elastic energy. Next, we describe the procedure of the elastic energy minimization leading to the optimal configurations of the HD. All the values defined in this section are related to the neutral planes of the monolayers, which are taken to be located at the interface between the regions of the monolayer polar heads and hydrophobic chains [4].

Monolayer deformations: tilt, splay and saddle splay and the elastic energy. Lipid tilt is generated as a result of slanting the average orientation of the hydrocarbon chains of lipid molecules with respect to the direction perpendicular to the monolayer surface. Equivalently, lipid tilt can be seen as shearing of the lipid hydrocarbon chains the direction perpendicular to the monolayer plane. Characterizing the lipid chain orientation by a unit vector, $\mathbf{n}$, referred below to as the lipid director, the lipid tilt can be defined as a deviation of, $\mathbf{n}$, from the unit vector of the surface normal, $\mathbf{N}$ [3],

$$\mathbf{t} = \frac{\mathbf{n}}{\mathbf{n}\cdot\mathbf{N}} - \mathbf{N}. \quad \text{(A1)}$$

From simple geometrical considerations it is easy to see that $|\mathbf{t}| = \tan\theta$, where θ is the angle between $\mathbf{N}$ and $\mathbf{n}$, called the tilt angle (Fig. A1 B).

The geometrical meaning of lipid splay is the two-dimensional divergence of the lipid director, $\boldsymbol{\nabla}\cdot\boldsymbol{n}$, defined along the curved monolayer surface. In covariant form, the lipid splay can be presented as a trace of a tensor, $\tilde{b}_\alpha^\beta = \nabla_\alpha n^\beta$, where the sub- and superscripts denote, respectively, the co- and contravariant components in the local coordinate basis of the surface [3]. Hence, $\boldsymbol{\nabla}\cdot\mathbf{n} = \nabla_\alpha n^\alpha = \tilde{b}_\alpha^\alpha$, where, according to the common convention, summation is performed over repeating indices. Assuming small tilt, $|\boldsymbol{t}| \ll 1$, the lipid director is, approximately, $\mathbf{n} \cong \mathbf{N} + \mathbf{t}$, and the lipid splay can be presented as [3],

$$\boldsymbol{\nabla}\cdot\mathbf{n} \cong \boldsymbol{\nabla}\cdot\mathbf{N} + \boldsymbol{\nabla}\cdot\mathbf{t}. \quad \text{(A2)}$$

The two-dimensional derivatives of the surface normal vector, $\mathbf{N}$, constitute the curvature

tensor, $b_\alpha^\beta = \nabla_\alpha N^\beta$ [5], whose trace, $\mathbf{\nabla} \cdot \mathbf{N} = \nabla_\alpha N^\alpha = b_\alpha^\alpha$, is referred to as the surface total curvature, J, or twice mean curvature [5]. The total curvature can be presented as the sum of the surface principal curvatures, $J = c_1 + c_2$ [5]. Based on these definitions, the lipid splay, $\mathbf{\nabla} \cdot \mathbf{n}$, is also referred to as the modified total curvature, $\tilde{J}$, accounting for the geometrical total curvature and the two-dimensional gradient of the tilt, $\tilde{J} = J + \mathbf{\nabla} \cdot \mathbf{t}$ [6]. The surface Gaussian curvature, K, is defined as the determinant of the curvature tensor, $K = \det b_\alpha^\beta$ [5]. The lipid saddle-splay, which is also referred to as the modified Gaussian curvature, $\widetilde{K}$, is a generalization of Gaussian curvature in the presence of both bending and tilt deformations, $\widetilde{K} = \det \tilde{b}_\alpha^\beta$ [3].

The energy of the tilt and saddle-splay deformations per unit area of the membrane plane is given by the generalized Helfrich model [3],

$$f_m = \frac{1}{2}\kappa_m\left(J + \mathbf{\nabla} \cdot \mathbf{t} - J_{0,m}\right)^2 - \frac{1}{2}\kappa_m J_{0,m}^2 + \bar{\kappa}_m \widetilde{K} + \frac{1}{2}\kappa_{m,t}\mathbf{t}^2, \qquad \text{(A3)}$$

where $J_{0,m}$ is the monolayer spontaneous splay, κ_m is the monolayer bending modulus, $\bar{\kappa}_m$ is the monolayer saddle splay modulus, and $\kappa_{m,t}$ is the monolayer tilt modulus.
The total elastic energy is calculated by integrating the energy density (Eq. A3) over the areas of the distal and proximal monolayers,

$$F = \int f_{m,d} dA + \int f_{m,p} dA, \qquad \text{(A4)}$$

where the subscripts d and p denote the distal and proximal monolayer, respectively.

Calculation of minimal energy configuration. To find the HD structure we minimize the elastic energy presented (Eq. A4). The fusion site is divided into three parts – the diaphragm, the proximal and distal monolayers of the peripheral zone (Fig. 2 A). The diaphragm is composed of the two distal monolayers that contact each other along a flat midplane. The distal and proximal monolayers of the peripheral zone, which correspond to the monolayers of the initial membranes, contact each other along a bent mid-plane (Fig. 2 A).

To minimize the total energy, we calculate separately the energy of the diaphragm, $F_{dia}(R_D, \varphi_0)$, and the energy of the monolayers of the peripheral zone, $F_{per}(R_D, \varphi_0)$, for different combinations of the diaphragm radius, R_D, and junction angel, φ_0. We require in our calculations that the lipid director, $\mathbf{n}$, and lipid tail length (Fig. 2 A), which is defined as a distance computed along the $\mathbf{n}$ direction between the position of a molecular polar head at the

neutral plane and the bilayer mid-surface, are continuous everywhere including the monolayer junction within the HD rim. With these restrictions and simple geometrical considerations, we find the tilt at the diaphragm rim to be $t_{0,d} = \tan\left[\frac{\pi}{4} - \frac{\varphi_0}{2}\right]$ in the distal monolayer and $t_{0,p} = \tan\varphi_0$ in the proximal monolayer. The minimal energy of the diaphragm, $F_{dia}(R_D, \varphi_0)$, is calculated in Appendix B (Appendix B, Eq. A16), the energy of the peripheral zone, $F_{per}(R_D, \varphi_0)$, is found using finite element analysis with the help of Surface Evolver [7]. The optimal configuration of R_D and φ_0 is found by minimizing the sum of the diaphragm and peripheral zone energy,

$$F_{opt} = \min\left[F_{per}(R_D, \varphi_0) + F_{dia}(R_D, \varphi_0)\right]. \quad \text{(A5)}$$

Assumptions and approximations. In our calculations κ_m, $\bar{\kappa}_m$ and $\kappa_{m,t}$ are assumed not to depend on the monolayer lipid composition and are identical everywhere, whereas the monolayer spontaneous curvature can be different for the distal and proximal monolayers. We simplified our analysis by neglecting the effects of the monolayer thickness variation due to the lipid splay gradients. In other words, the monolayer dividing plane was considered to be parallel to the membrane mid-plane everywhere. The lipid tail length was related to the tilt by the relationship $= \delta_0\sqrt{1 + \mathbf{t}^2}$, which follows from the requirement of a fixed volume per lipid molecule upon a possibility of expansion of the in-plane molecular lipid area. The energy contribution of the lipid area expansion is proportional to the square of gradient splay and is negligible according to our estimations. Finally, we neglected the contribution of the saddle splay while minimizing the energy, but took it into account by computing the energy barrier of the pore formation, which is justified in Appendix E.

Appendix B

Lateral tension and elastic energy of the diaphragm

Here we find the lateral tension, $\gamma(R_D, \varphi_0)$, and the elastic energy of the diaphragm, $F_{dia}(R_D, \varphi_0)$, as functions of the diaphragm radius R_D and the junction angle φ_0. We start with explicit description of the tilt, splay, and saddle-splay in the diaphragm. Next, we minimize the elastic energy with respect to the diaphragm radius R_D and the junction angle φ_0. Finally, we find the minimal diaphragm energy and the corresponding lateral tension.

Tilt, splay and saddle splay in the diaphragm. The symmetry of the HD and the geometrical constraint at its boundary suggest that the lipid director must point towards the center of the diaphragm (Fig. A1), therefore, tilt vector (Eq. A1) in the diaphragm can is expressed as

$$\mathbf{t} = -t(r)\hat{\mathbf{r}}, \tag{A6}$$

where $\hat{r}$ is the unit radial vector and $t(r)$ is the magnitude of tilt at a distance r from diaphragm center (see Fig. A1). The monolayers of the HD are flat, $J = 0$, so that the expression for the lipid splay (Eq. A2) is simplified to $\tilde{J} = \mathbf{\nabla} \cdot \mathbf{t}$. In cylindrical coordinate system the expression for splay is,

$$\tilde{J} = \mathbf{\nabla} \cdot \mathbf{t} = -\frac{\partial t}{\partial r} - \frac{t}{r}. \tag{A7}$$

We refer to the first and second terms in (Eq. A7) as the meridional and parallel splay, respectively. The saddle splay can be presented as a product of the meridional and parallel splays:

$$\widetilde{K} = \frac{\partial t}{\partial r} \cdot \frac{t}{r}. \tag{A8}$$

Minimization of the elastic energy in the diaphragm. Next, we proceed to finding the minimal energy configuration of the diaphragm monolayers by using the regular procedure based on the variational principle. Tilt is perturbed by a small increment, $\boldsymbol{t} \to \boldsymbol{t} + \delta\boldsymbol{t}$, while keeping the geometrical shape of the monolayer fixed. The tilt at the rim of the diaphragm is given by the junction geometry, so that its variation is required to vanish, $\delta\boldsymbol{t}_{r=R_D} = 0$. To find the equilibrium tilt distribution in a given monolayer, we first find the resulting variation in energy density of a monolayer, δf, and than the total variation of the monolayer elastic energy $\delta F = \int \delta f \, dA$. From the requirement that $\delta F = 0$ we can derive a differential equation for the tilt distribution, which is Euler-Lagrange (EL) equation. To simplify the analysis, we neglect at this stage the contribution to the energy by the saddle splay deformation, which is justified in Appendix E. Such EL equation was derived in [6], and can be represented as

$$r^2 \frac{\partial^2 t}{\partial r^2} + r\frac{\partial t}{\partial r} = (1 + r^2 l^{-2})t. \tag{A9}$$

This is Sturm-Liouville differential equation and its general solution is

$$t(r) = C_1 \mathrm{I}_1(l^{-1}r) + C_2 \mathrm{K}_1(l^{-1}r), \tag{A10}$$

where I and K are the modified Bessel functions of the first and second kind, respectively, and C_1, C_2 are integration constants.

Diaphragm energy and lateral tension. We use the boundary conditions in the diaphragm rim and center. As mentioned in Appendix A, the tilt of the distal monolayer for, $r = R_D$, is related to the junction angle by $t(r = R_D) = \tan\left[\frac{\pi}{4} - \frac{\varphi_0}{2}\right]$. Based on the rotational symmetry of the diaphragm, tilt must vanish at the center of the diaphragm, $t(r = 0) = 0$. Using these two boundaries conditions we can find the integration constants (Eq. A10) to be

$$C_2 = 0 \quad , \quad C_1 = -\frac{\tan\left[\frac{\pi}{4} - \frac{\varphi_0}{2}\right]}{\mathrm{I}_1(l^{-1}R_D)} . \tag{A11}$$

Hence, the tilt at any point along the diaphragm (Fig. A2 A) is given by:

$$t_{dia}(r) = -\tan\left[\frac{\pi}{4} - \frac{\varphi_0}{2}\right]\frac{\mathrm{I}_1(l^{-1}r)}{\mathrm{I}_1(l^{-1}R_D)} . \tag{A12}$$

Using Eqs. A7 and A8 (Fig. A2 B and C) we obtain that lipid splay and saddle-splay in the diaphragm are given, respectively, by

$$\tilde{J}_{dia}(r) = -\frac{\tan\left[\frac{\pi}{4} - \frac{\phi_0}{2}\right]}{2l\mathrm{I}_1(l^{-1}R_D)}\left[\mathrm{I}_0(l^{-1}r) + 2\frac{l}{r}\mathrm{I}_1(l^{-1}r) + \mathrm{I}_2(l^{-1}r)\right], \tag{A13}$$

$$\tilde{K}_{\mathrm{dia}}(r) = \frac{t}{r}\frac{\partial t}{\partial r} = \frac{\tan^2\left[\frac{\pi}{4} - \frac{\phi_0}{2}\right]}{2rl}\frac{\mathrm{I}_1(l^{-1}r)}{\mathrm{I}_1^2(l^{-1}R_D)}\left[\mathrm{I}_2(l^{-1}r) + \mathrm{I}_0(l^{-1}r)\right]. \tag{A14}$$

The membrane lateral tension is equal to monolayer energy per unit area, which, according to (Eq. A3) is given by:

$$\gamma(r) = 2f_m = \kappa_m\left(\tilde{J}_{dia} - J_{0,m}\right)^2 - \kappa_m J_{0,m}^2 + 2\bar{\kappa}_m\tilde{K}_{dia} + \kappa_{m,t}t_{dia}^2, \tag{A15}$$

substituted with (Eqs. A3, A12, A13 and A14) (Fig. A2 D). The total elastic energy of the diaphragm is given by integration of (Eq. A15) over the entire diaphragm area:

$$F_d = 2\pi\int_{r=0}^{r=R_D}\gamma(r)rdr . \tag{A16}$$

Example of lipid tilt, splay, saddle-splay and lateral tension radial distribution in the diaphragm is presented in Fig. A2.

Appendix C

Energy barrier and the rate of fusion pore formation

Here we derive the expression for the energy barrier and the characteristic time of the fusion pore formation. A model for pore formation in a flat lipid membrane with uniform lateral tension was introduced and explored in a series of works such as in [8]. The essence of the model is an interplay between the membrane lateral tension, γ, and the energy of the pore rim

related to the rim unit length and referred to as the pore line tension, λ. The pore growth is favored by the energy gain due to the membrane removal from the stressed region and counteracted by the energy cost of the pore rim expansion. The pore behavior is determined by the relationship between the pore radius, ρ, and its critical value, ρ^*. Pores with $\rho < \rho^*$ tend to close while pores with $\rho > \rho^*$ tend to infinitely expand. The activation energy for pore formation, E^*, referred to by us as barrier energy, is the energy of pore with the critical pore radius, ρ^*. The characteristic time of formation of a large fusion pore , τ, in a membrane with area A is given by [8]

$$\tau = \frac{A_0\tau_0}{A}\, e^{\frac{E^*}{k_bT}}, \tag{A17}$$

where k_b is the Boltzmann constant, and T is the absolute temperature. A_0 and τ_0 are two microscopic constants with units of area and time, respectively, that are unknown but can be assumed not to change upon small variations of the membrane lipid composition.

To calculate the energy barrier, E^*, we modify the model proposed by Taupin et. al. [8] to account for changes of the lateral tension along the membrane plane related to the tilt and splay gradients. The energy gained by removal of the membrane material from the stressed bilayer will be calculated as $\int \gamma dA$, where γ membrane lateral tension given in the diaphragm by (Eq. A15) in Appendix B. The pore line tension, λ, will be assumed to be independent of the pore radius, as proposed in [8].

Since, according to our computations, the lateral tension is larger near the rim than in the middle of the diaphragm, a pore is expected to start forming in close to the rim. However, according to our computations the critical pore size is comparable to the diaphragm radius. Hence, before reaching the critical size, which corresponds to its maximal energy and determines, therefore, the energy barrier, the pore spreads to most of the diaphragm. As a result, the location of the point of the pore initiation, practically, does not change the value of the energy barrier. Therefore, in our calculations we assume, for simplicity, that the pore starts forming at the center of the diaphragm and expands radially. Since the lateral tension is lower at the center, this assumption leads to some overestimation of the energy barrier, and, thus, to an overestimation of the pore formation time.

Further, we assume that the pore formation is a fast process, so that the HD shape does not change during the pore expansion. The pore expansion is accompanied by exchange of lipid

monolayer area between the stressed diaphragm and the surrounding membrane playing the role of a lipid reservoir. The energy balance can be presented as:

$$E_{pore}(\rho) = 2\pi\rho\lambda - 2\pi \int_{r=0}^{r=\rho} \gamma(r) r dr, \qquad \text{(A18)}$$

where $\gamma(r)$ is given by (Eq. A15) in Appendix B. The barrier energy is the maximum of Eq. A18,

$$E^* = \max[E_{pore}(\rho)]. \qquad \text{(A19)}$$

Finally, we define the acceleration factor of the pore formation, β, as the ratio between the characteristic times of pore formation in the initial, τ_i, and final, τ_f, states of the diaphragm, $\beta = \frac{\tau_i}{\tau_f}$. The acceleration factor is given by

$$\beta = \frac{\tau_i}{\tau_f} = \frac{A_f}{A_i} e^{\frac{E_i^* - E_f^*}{k_b T}}, \qquad \text{(A20)}$$

where A_i and A_f are the diaphragm areas in the initial and final states, E_i^* and E_f^* are the barrier energies calculated using (Eq. A19) for the initial and final states.

Appendix D

The effect of changing the fusion site dimension

In the main text we assumed that the radius of the fusion site, R_B has a fixed value set by the fusion machinery that drives the fusion process. Here we explore another scenario in which the size of the fusion site is free to change, but the distance between the diaphragm rim, R_D, and the fusion site boundary, $y^* = R_B - R_D$, remains constant (Fig. 2A). We will call this scenario the "free boundary" regime, whereas the scenario presented in the main text will be referred to as the "fixed boundary" regime.

Our computations showed that the model predictions for the "free boundary" regime strongly depend on the characteristic length of tilt relaxation, $l = \sqrt{\frac{\kappa_t}{\kappa_m}}$, where κ_t and κ_m are, respectively, the monolayer tilt and splay (bending) moduli [9].

At short decay length l, the acceleration factor is predicted similar values for the "free boundary" and "fixed boundary" scenarios, while at long tilt decay length the predictions for the two scenarios are different (Fig. A3 B Vs. Fig. 3E). We explored, computationally, the effect on the acceleration factor of fusion pore formation, β, produced by addition to the proximal monolayers of LPC in concentrations corresponding to up to 10% area fraction. In the "free boundary" scenario, for tilt decay length $= 1$ nm , resulted in the acceleration factor of up to 1.2 (Fig. A3 B). For membrane with tilt decay length $l = 1.5$ nm the acceleration factor had values up to $2.5 - 3$ (Fig. A3 B). And, finally, for long tilt decay length of, $l = 2$ nm, the effect of the proximal monolayer spontaneous curvature was predicted to be very substantial such that even small changes of J_s^{out} corresponding to the added LPC area fraction of just 3% were predicted to accelerate the fusion pore formation by a factor about 100 (Fig. A3 B).

Appendix E

The effects of lipid redistribution

The elastic energy and the stress-related phenomena can be reduced by the lipid redistribution between the membrane bulk and the elastically stressed regions of the membrane monolayers. The regions of lipid monolayers forming the HD rim are characterized by a substantially negative splay of lipid molecules. Therefore, depletion of lipid molecules with positive molecular curvature from and enrichment of lipid molecules with negative molecular curvature in the rim region are expected to decrease the local stress and, consequently, reduce, to some extent, the acceleration factor of the fusion pore formation. To evaluate this effect, one has to consider that the lipid enrichment and/or depletion in certain regions are counteracted by the entropy, which favors an even distribution of all lipid species across the whole plane of each monolayer. Therefore, the lipid repartitioning is expected to be substantial only if the resulting relaxation of the elastic energy related to one lipid molecule is comparable to or exceeds the thermal energy $k_B T$ (the product of the Boltzmann constant and the absolute temperature). The area occupied by one lipid molecule in the membrane plane is rather small and, as a result, the elastic energy relaxation is, typically, smaller than $k_B T$, so that the effects of the lipid redistribution are small.

This said, we estimated the effect of the lipid redistribution on the acceleration factor predicted by our model. As a relevant example we considered the depletion from the rim region of LPC molecules characterized by a large molecular curvature, $\xi_{LPC} = 0.26$ nm^{-1}. Following a similar derivation procedure as in [10] we found that the local mole fraction of LPC, ϕ_{LPC}^{p}, within the

rim region characterized by the average local lipid splay, $\tilde{J}$, is related to the mole fraction of this lipid in the membrane bulk, ϕ^{r}_{LPC}, by,

$$\phi^{p}_{LPC} = \frac{\phi^{r}_{LPC}}{(1-\phi^{r}_{LPC})\exp\left(-\kappa_m \tilde{J} a \frac{\xi_{LPC}-\xi_0}{k_b T}\right) + \phi^{r}_{LPC}}. \quad \text{(A21)}$$

In this expression, a is the area per lipid molecule in the membrane plane, which we assumed identical for all lipids, ξ_0 is the molecular curvature of the background lipid, and κ_m is the bending modulus of a lipid monolayer. For estimation, we used the typical parameter values, $\kappa_m = 10\mathrm{k_bT}$, [11], $\xi_0 = -0.1$ nm^{-1} [12, 13], $a = 0.7$ nm^2 [11-13]. For the sake of a conservative estimation, we used the upper limit of the considered range of LPC bulk mole ratios, $\phi^{r}_{LPC} = 0.1$. According to our calculations, the splay in the proximal monolayer has the most negative value near the HD rim and decays to negligible values within a distance of a few nanometers. Assuming the characteristic decay length of deformations to equal $l = 1.5nm$, we calculated the mean splay to be $\tilde{J} \sim -0.1$ nm^{-1}. Using this value for $\tilde{J}$, and assuming the typical geometrical characteristics of the fusion site to be $R_B = 15$ nm, and $x^* = 6.6$ nm, the pore line tension and the saddle splay modulus to constitute $\lambda = 10$ pN and $\bar{\kappa}_m = -5\mathrm{k_bT}$, respectively, we found the local mole fraction of LPC around the rim to be $\phi^{p}_{LPC} \sim 0.08$. The acceleration factor was computed to be reduced from $\beta = 8.3$ before allowing LPC redistribution to $\beta = 6.9$ upon the redistribution. This means that both the local LPC concentration in the rim region and the acceleration factor decreased by about 20%. Such correction does not change the qualitative conclusions of our work.

Appendix F

Effects of the saddle splay modulus and pore line tension

We distinguish between two effects of the saddle splay on the energy barrier and the acceleration factor of the fusion pore formation: a direct effect related to the energy density, $f_{\tilde{K}} = \bar{\kappa}_m \tilde{K}$, and an indirect effect originating from the changes of the monolayer conformations related to minimization of the saddle splay energy. In the calculations whose results are presented in the main text, we accounted only for the direct effect while the indirect one was neglected. In the following, we show that the indirect effect can only accelerate the pore formation, so that our results can be considered as underestimation of the real fusion acceleration factor.

The saddle splay at the diaphragm is the product of the two principle components $\widetilde{K}_{diph} = \frac{t}{r}\frac{\partial t}{\partial r}$ (Eq. A8). The contribution to the elastic energy associated with the saddle splay, $F_{\widetilde{K}}$, is given by integrating over monolayer area:

$$F_{\widetilde{K}} = \int f_{\widetilde{K}}\, dA = 2\pi\bar{\kappa}_m \int_{r=0}^{r=R_D} t \frac{\partial t}{\partial r} dr = \pi\bar{\kappa}_m t^2(r = R) < 0, \qquad \text{(A22)}$$

where the integration is performed over the areas of the diaphragm monolayers.

Since saddle splay modulus $\bar{\kappa}_m$ has negative value [14, 15] the contribution of $F_{\widetilde{K}}$ (Eq. A22) to the total elastic energy is negative and independent of the diaphragm radius. It depends only on the tilt at the diaphragm rim. Therefore, accounting for this contribution (Eq. A22) in the energy minimization does not change the radius of the diaphragm but decreases the junction angle φ_0 (Fig. 2 A) and, hence, increases the tilt at the diaphragm. This results in higher lateral tension and larger rates of the fusion pore formation.

The direct effect of the monolayer saddle splay modulus, $\bar{\kappa}_m$, on the acceleration factor produced by the positive spontaneous curvature of the proximal monolayers, J_S^0, is illustrated in (Fig. A4).

The pore line tension, λ, has a significant effect on the energy barrier and the acceleration factor for fusion pore opening. We calculated this effect for the line tension range of $10 - 30$ pN corresponding to the data currently available in the literature for DOPC membrane [16]. The results are presented in Fig. A5. In all the calculation results presented in the main text, we took the lower limit of the range, $\lambda = 10$ pN, to get an under-estimation of the real acceleration factor. If we use more feasible pore line tension, $\lambda = 20$ pN the acceleration factor increases from $\beta = 4$ to $\beta = 48$ upon addition to the proximal monolayers of LPC in concentration corresponding to 7.5% area fraction. Using the highest possible value of λ, we obtained the acceleration factor of $\beta = 766$.

Appendix references

[1] Y. Kozlovsky and M. M. Kozlov, "Stalk model of membrane fusion: solution of energy crisis," *Biophysical journal,* vol. 82, no. 2, pp. 882-895, 2002.

[2] Y. Kozlovsky, L. V. Chernomordik, and M. M. Kozlov, "Lipid intermediates in membrane fusion: formation, structure, and decay of hemifusion diaphragm," *Biophysical journal,* vol. 83, no. 5, pp. 2634-2651, 2002.

[3] M. Hamm and M. M. Kozlov, "Elastic energy of tilt and bending of fluid membranes," *The European Physical Journal E,* vol. 3, no. 4, pp. 323-335, 2000.

[4] M. Kozlov and M. Winterhalter, "Elastic moduli for strongly curved monoplayers. Position of the neutral surface," *Journal de Physique II,* vol. 1, no. 9, pp. 1077-1084, 1991.

[5] M. M. Kozlov, "Membrane shape equations," *Journal of Physics: Condensed Matter,* vol. 18, no. 28, p. S1177, 2006.

[6] M. M. Terzi and M. Deserno, "Novel tilt-curvature coupling in lipid membranes," *The Journal of chemical physics,* vol. 147, no. 8, p. 084702, 2017.

[7] K. A. Brakke, "The Surface Evolver," *Experimental Mathematics,* vol. 1, no. 2, pp. 141-165, 1992/01/01 1992, doi: 10.1080/10586458.1992.10504253.

[8] C. Taupin, M. Dvolaitzky, and C. Sauterey, "Osmotic pressure-induced pores in phospholipid vesicles," *Biochemistry,* vol. 14, no. 21, pp. 4771-4775, 1975.

[9] M. Hamm and M. Kozlov, "Tilt model of inverted amphiphilic mesophases," *The European Physical Journal B-Condensed Matter and Complex Systems,* vol. 6, no. 4, pp. 519-528, 1998.

[10] V. Choudhary *et al.*, "Architecture of lipid droplets in endoplasmic reticulum is determined by phospholipid intrinsic curvature," *Current Biology,* vol. 28, no. 6, pp. 915-926. e9, 2018.

[11] N. Fuller and R. P. Rand, "The influence of lysolipids on the spontaneous curvature and bending elasticity of phospholipid membranes," (in English), *Biophysical Journal,* Article vol. 81, no. 1, pp. 243-254, Jul 2001.

[12] J. A. Szule, N. L. Fuller, and R. P. Rand, "The effects of acyl chain length and saturation of diacylglycerols and phosphatidylcholines on membrane monolayer curvature," *Biophys J,* vol. 83, no. 2, pp. 977-84, Aug 2002.

[13] Z. Chen and R. P. Rand, "The influence of cholesterol on phospholipid membrane curvature and bending elasticity," *Biophys J,* vol. 73, no. 1, pp. 267-76, Jul 1997, doi: 10.1016/S0006-3495(97)78067-6.

[14] R. H. Templer, B. J. Khoo, and J. M. Seddon, "Gaussian curvature modulus of an amphiphilic monolayer," *Langmuir,* vol. 14, no. 26, pp. 7427-7434, 1998.

[15] M. M. Terzi, M. F. Ergüder, and M. Deserno, "A consistent quadratic curvature-tilt theory for fluid lipid membranes," *The Journal of chemical physics,* vol. 151, no. 16, p. 164108, 2019.

[16] T. Portet and R. Dimova, "A new method for measuring edge tensions and stability of lipid bilayers: effect of membrane composition," *Biophysical journal,* vol. 99, no. 10, pp. 3264-3273, 2010.

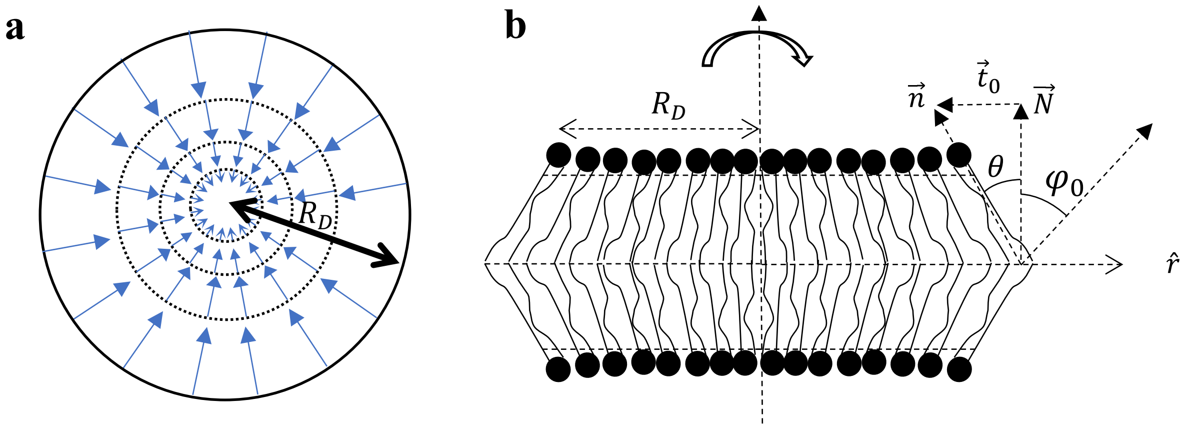

Figure A1

Distribution of the tilt deformation in the diaphragm. (A) The upper view of the diaphragm. The arrows indicate the tilt direction and absolute value. (B) Schematic presentation of the diaphragm cross-section illustrating the definitions of the lipid director, $\vec{n}$, the normal unit vector diaphragm, $\mathbf{N}$, the tilt vector, $\mathbf{t}$, the tilt angle, θ, and the junction angle φ_0, as presented in Fig. 2 A.

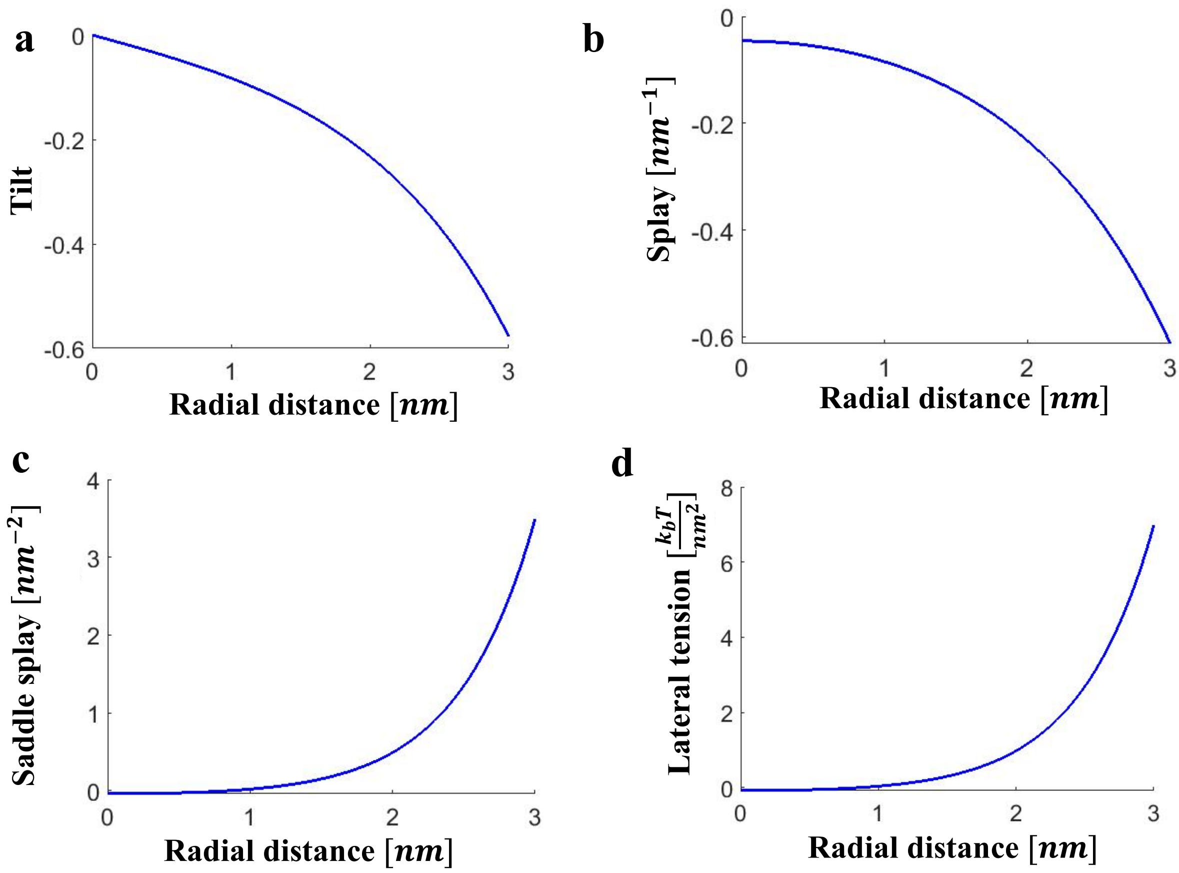

Figure A2

Distribution of the monolayer deformations in the diaphragm: (A) lipid tilt, (B) lipid splay, (C) lipid saddle splay, (D) lateral tension for parameter values, $\kappa_m = 10\mathrm{k_b T}$, $\kappa_t = 40\,\frac{\mathrm{mN}}{\mathrm{m}}$, $\tilde{\kappa}_m = 0$, $R_D = 3$ nm, $t_0 = \tan\frac{\pi}{6}$, and $J_{sm} = -0.1$ nm^{-1}.

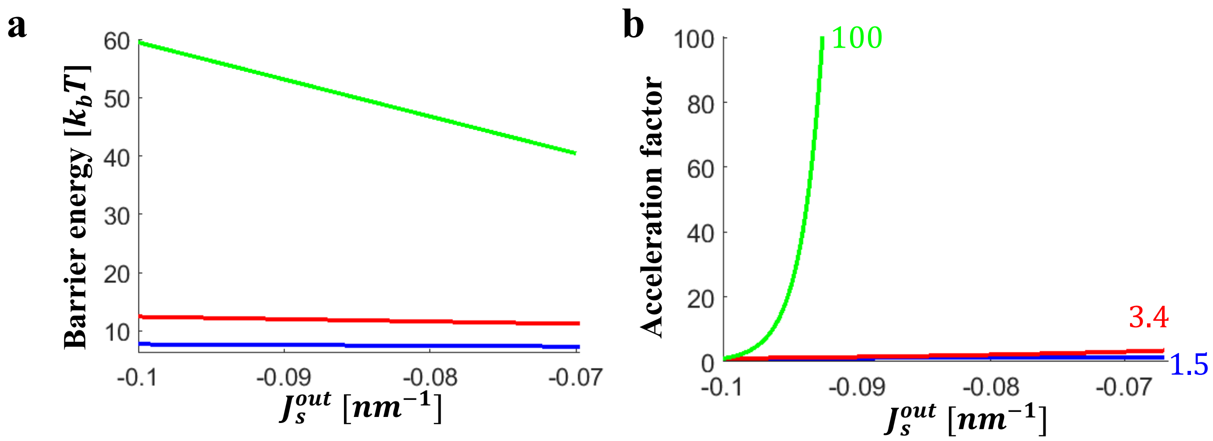

Figure A3

Effect of tilt relaxation length, l, in the "free boundary" scenario on the dependence of (A) the energy barrier and (B) the acceleration factor of the fusion pore formation on the spontaneous curvature of the proximal monolayers. Blue $l = 1$ nm, red $l = 1.5$ nm, green $l = 2$ nm. The parameter values: $x^* = 6.6$ nm, $y^* = 15$ nm, $\kappa_m = 10k_bT$, $J_S^{in} = -0.1$nm^{-1}, $\bar{\kappa}_m = -5k_bT$, $\lambda = 10$ pN. Acceleration factor for maximal calculated amount of LPC indicated in (B): Blue line ($l = 1$ nm) maximal amount of LPC is 10% area fraction, $\beta = 1.5$. Red line ($l = 1.5$ nm) maximal amount of LPC is 10% area fraction, $\beta = 3.4$. Green line ($l = 2$ nm) maximal amount of LPC in proximal monolayers is 2.2% area fraction, $\beta = 100$.

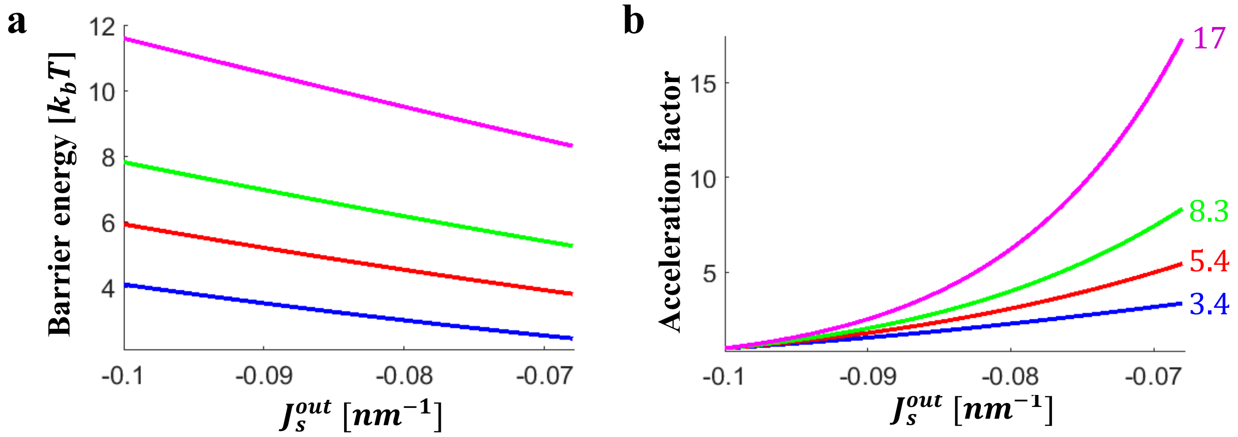

Figure A4

Effect of saddle splay modulus on the dependence of the (A) the energy barrier and (B) the acceleration factor of the fusion pore formation on the spontaneous curvature of the proximal monolayers. Magenta $\bar{\kappa}_m/\kappa_m = -1$, green $\bar{\kappa}_m/\kappa_m = -0.5$, red $\bar{\kappa}_m/\kappa_m = -0.25$, blue $\bar{\kappa}_m/\kappa_m = 0$. The parameter values: $x^* = 4.6$ nm, $\kappa_m = 10k_bT$, $l = 1.5$ nm, $R_B = 15$ nm, $\lambda = 10$ pN. Acceleration factor for 10% area fraction of LPC added to the proximal monolayers as indicated in (B).

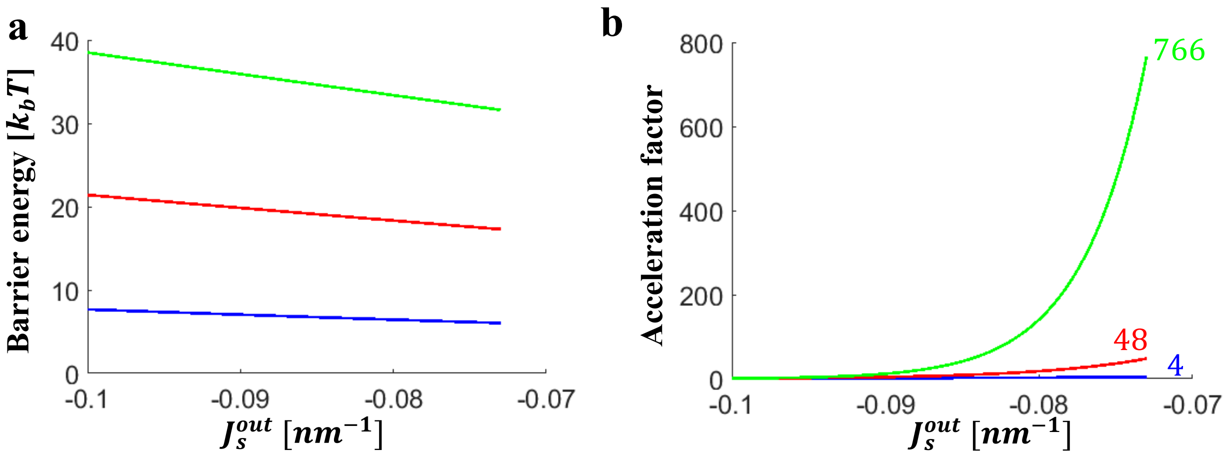

Figure A5

Effect of the pore line tension on the dependence of the (A) the energy barrier and (B) the acceleration factor of the fusion pore formation on the spontaneous curvature of the proximal monolayers. Blue $\lambda = 10$ pN, red $\lambda = 20$ pN, green $\lambda = 30$ pN. The parameter values: $x^* =$ 6.6 nm, $R_B = 15$ nm, $\kappa_m = 10 k_b T$, $J_S^{in} = -0.1$ nm^{-1}, $\bar{\kappa}_m = -5 k_b T$, $l = 1.5$ nm. Acceleration factor for 7.5% area fraction of LPC added to the proximal monolayers as indicated in (B).
